# Patient Advocates for Clinical Research (PACER): A Step Toward Ethical, Relevant, and Truly Participatory Clinical Research in India

**DOI:** 10.7759/cureus.58454

**Published:** 2024-04-17

**Authors:** Poonam Bagai, Pooja Sharma, Aala Ansari, Nirbhay Singh, Sonal Sharma, Padam Singh, Durga Chougule, Manish Kumar Singh, Gargi Singh, Sanjeev Singh

**Affiliations:** 1 Pediatric Cancer Research Institute, CanKids KidsCan, New Delhi, IND; 2 Obstetrics and Gynecology, APAR Health, Gurugram, IND; 3 Patient Navigation, Advocacy, and Family Engagement, CanKids KidsCan, New Delhi, IND; 4 Clinical Research, Medanta Institute of Education and Research, Gurugram, IND; 5 Amrita Institute of Medical Sciences, Amrita Hospital, Faridabad, IND

**Keywords:** focus group discussion, participants’ rights, ethics, good clinical practice, patient advocacy group, clinical research

## Abstract

Background

Clinical research presents a promising path for improving healthcare in contemporary India. Yet, researchers identify gaps in trust, awareness, as well as misconceptions about being a ‘“guinea pig.” We proposed building the capacity of training patient advocacy groups (PAGs) in patient-centered clinical research and through them creating aware patients as research partners.

Methodology

Patient Advocates for Clinical Research (PACER) is a tiered program to share information and education about clinical research with PAGs. Tier one is a self-paced online learning course, followed by workshops on clinical research, Good Clinical Practice, research consent, case studies, and group discussions.

Results

A total of 20 PAGs represented by 48 participants, active in areas of pediatric cancer, breast cancer, multiple myeloma, type I diabetes, spinal muscular atrophy, sickle cell disease, and inflammatory bowel diseases, participated. Among 48 participants 30 successfully completed the online course (multiple-choice question evaluation score cut-off >70%), attaining an average score of 23.9 ± 2.1 out of 30. Overall, 48 participants attended workshop 1 and 45 workshop 2, with 140 participants joining the focus group discussion (FGD). An overall improvement of 9.4% (𝜒^2^ = 46.173; p < 0.001) for workshop 1 and 8.2% (𝜒^2^ = 25.412; p < 0.001) for workshop 2 was seen in knowledge gain about clinical research. The FGD raised issues such as misleading information from research teams, unethical recruitment, incomprehensible information sheets, and limited trial-related knowledge fostering fear of participation in clinical research.

Conclusions

Multimodal and tiered learning of clinical research such as that used by PACER has a good participatory and learning response from PAGs and may be further explored.

## Introduction

Clinical research is not only the route to drug development but also for improving access to care and quality of care [1-3]. Earlier, patients participated in clinical trials as “subjects” and had limited knowledge about the trials they were enrolled in. However, the perspective has since changed from a “subject” to being a “part” of the trials, i.e., a “participant” [4]. This change is also seen in in the earlier paternalistic doctor-patient relationship to the current patient-centered medicine model with “nothing about me without me” [5,6]. Critical to note that patient-centered medicine is not complete until patients participate not only in their own healthcare decisions but also in the research that informs such decisions [7]. Patient advocates or patient advocacy groups (PAGs) play a major role in patient engagement in clinical research [8-10].

Globally, the World Health Organization (WHO) seeks to engage patients and families, enabling collaborative partnerships worldwide toward fostering safer healthcare services through ‘“A Decade of Patient Safety 2021-2030.” Clinical Trials Transformation Initiative (CTTI), a joint effort between Duke University and the US Food and Drug Administration (USFDA), acknowledges patient groups as vital collaborators in clinical research [11-15]. The Patient-Centered Outcomes Research Institute in the United States [16] and the James Lind Alliance in the United Kingdom [17] have specific initiatives on information and education of participants, identification of research priorities, leading and designing research, improving access to clinical trials, and assessing patients’ experience. The implementation of these initiatives hopes to empower the development of an “expert patient” who will be actively involved in all the activities [18].

India has become the hub of clinical trials as well as the Asian hub of innovation and technology in healthcare [19-21]. In the last decade, many changes have been made in the Indian regulatory framework to enhance patient protection and centricity [22-26].

Yet, distrust, lack of awareness, perceptions, and consequent attitudes toward clinical research have contributed to low clinical trial participation [27-33]. PAGs convey the concerns and perspectives of patients to healthcare partners, and their involvement will raise the positive perception of clinical trials among the public and ensure patient-centered study design [34].

Patient Advocates for Clinical Research (PACER) initiative is an exploratory framework for information and education and building capacity of PAGs about clinical research in India. This study focuses on the strategies used to engage them in clinical research understanding and their outcomes.

## Materials and methods

The PACER India project was conceptualized for pan-India one year, from January 2023 to January 2024. The study was approved by the CanKids Ethics Committee (approval number: IEC-CK-2023-03). A waiver of consent was granted due to the minimal-risk nature of the study and the anonymized collection of data through the questionnaire.

Population

The target population involved with the program were PAGs working in the field of pediatric cancer, breast cancer, multiple myeloma, type I diabetes, spinal muscular atrophy, sickle cell disease, and inflammatory bowel diseases. The program reached out to 50 PAGs/patient advocates through online meetings, of which 20 groups enrolled themselves in the program.

Intervention

A tiered education program with a self-paced, simple language, online course, followed by quarterly half-day workshops with focus group discussions (FGDs) comprised the intervention.

PACER online course

The course included the following pre-recorded video modules: Module 1 - Why should patients participate in clinical research?; Module 2 - Ethics in clinical research. Module 3 - Orientation to clinical research; Module 4 - Indian Good Clinical Practice (GCP) course, including consent, compensation, and insurance; and Module 5 - Patient education about research methods

PACER workshops

Two workshops with in-person as well as virtual participation were conducted at an interval of 2.5 months. In addition to a lecture series, the workshops also served as a platform for small and large group discussion with a review of consent forms for observational studies, registries, and clinical trials, enabling the exchange of ideas and sharing of experiences and perspectives of participants. The effectiveness of the workshops on clinical research knowledge gain among participants was evaluated through pre- and post-workshop questionnaires. The purpose of the questionnaires and the confidentiality of the acquired data were explained to participants at the beginning of the workshops.

The first workshop was conducted to raise awareness of clinical research and its importance in advancing medical knowledge and improving patient outcomes among PAGs. The topics covered in this workshop emphasized “what is clinical research?,” “importance of informed consent,” “governance of clinical research by ethical, legal, and regulatory frameworks,” and “importance of clinical research for patients and patient advocates.”

The second workshop emphasized GCP and research ethics involved in clinical research and its technicalities.

The FGD held after the second PACER workshop was themed on patients, survivors, and caregivers’ active engagement in discussion and sharing their personal experiences, perceptions, and concerns on clinical research. This FGD was an online event and the participants were recruited from 50 PAGs through online invitations. A total of 140 participants comprising patients, survivors, and patient advocates attended the FGD.

Comparison

The success of dissemination of information among the PAGs was assessed through quiz scoring for the PACER course, whereas pre- and post-questionnaire evaluations containing 10 questions were assessed for the workshops.

Outcomes

Online Course

Learning management system-based and multiple-choice question (MCQ)-based evaluation was conducted (course completion certification was given to those with a score >70% on the MCQ).

Workshops

**Figure 1:**
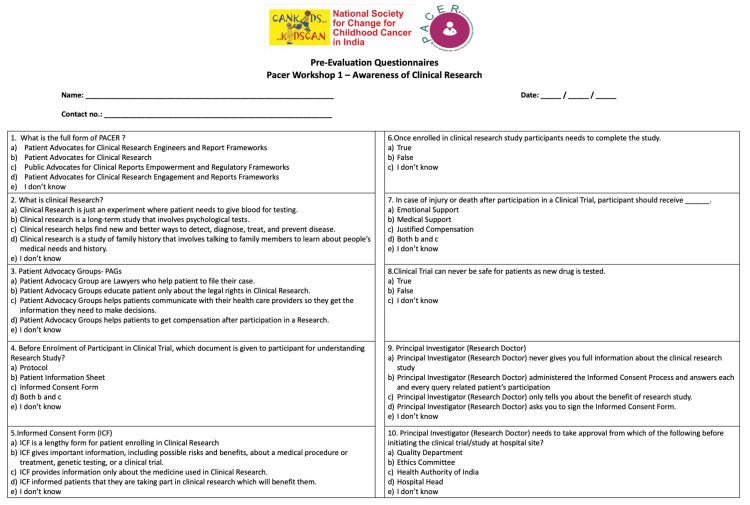
Pre-evaluation questionnaire PACER Workshop 1: Awareness of clinical research. PACER = Patient Advocates for Clinical Research

**Figure 2:**
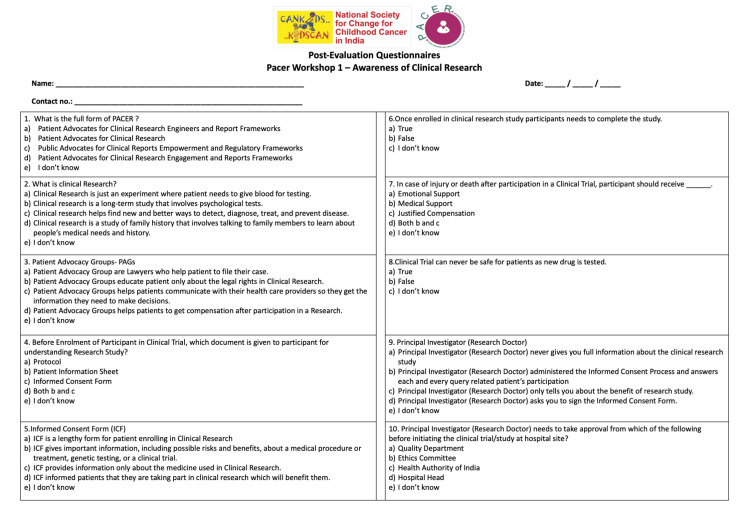
Post-evaluation questionnaires PACER Workshop 1: Awareness of clinical research. PACER = Patient Advocates for Clinical Research

**Figure 3:**
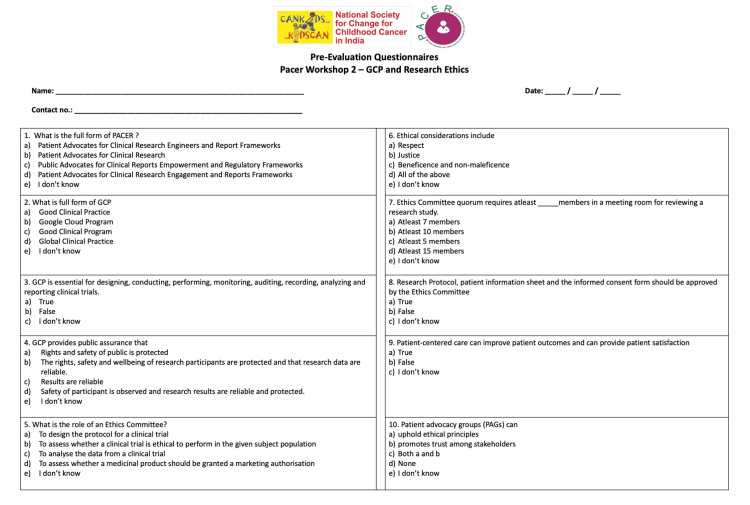
Pre-evaluation questionnaires PACER Workshop 2: GCP and research ethics. PACER = Patient Advocates for Clinical Research; GCP = Good Clinical Practice

**Figure 4:**
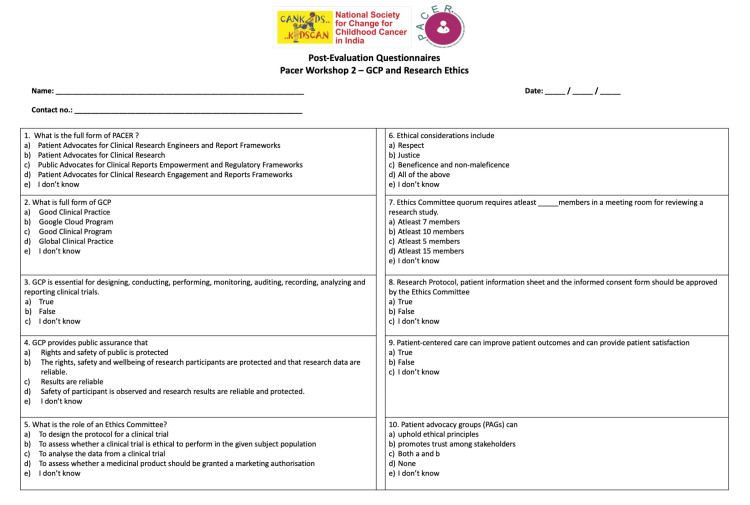
Post-evaluation questionnaires PACER Workshop 2: GCP and research ethics. PACER = Patient Advocates for Clinical Research; GCP = Good Clinical Practice

Pre- and post-evaluation questionnaires (Appendices, Figures 1-4) were disseminated to assess workshop interaction.

Through this educational program, information about the conduct of clinical research and the rights of participants during their enrolment in studies will be disseminated among PAGs. The orientation of PAGs will develop a positive outlook toward clinical research and increase awareness among the public, thus improving the holistic care for patients.

Statistical analysis

The response to each question both pre and post is presented as frequency and percentage. The overall score for pre and post-combining all questions is presented as frequency and percentage. The online PACER course score combining all questions is represented as mean ± SD, with a score of 1 for a right answer and 0 for a wrong answer. For statistical analysis of improvement in correct responses to different questions, the McNemar test was used. Importantly, this statistical test of significance is appropriate to analyze paired categorical data, typically for the pre-post or before-after comparisons. Statistical significance was considered at p-values <0.05. The data analysis was done using SPSS version 26.0 (IBM Corp., Armonk, NY, USA).

## Results

A total of 20 PAGs, represented by 48 people, actively participated in the PACER program. The online course included pre-recorded video modules on “Why patients should participate in clinical research?,” “Bioethics,” “Clinical Research in India,” “Good Clinical Practice,” and “Research methods.”

Overall, 30 of the 48 who were offered the course successfully completed it, with an average score of 23.9 ± 2.1 (range = 21-29) of a total score of 30. The other 18 who partially completed the course were prompted to complete it over one year.

Workshops

Workshop 1 on awareness about clinical research was attended by 48 participants on March 31, 2023. All attendees had previously reviewed the online course material. The participants were assessed on their familiarity with the terms “PACER,” “clinical research,” and “patient advocacy groups.”

The success of patient-centered studies relies on well-informed patients familiar with ethical, legal, and regulatory frameworks, enabling them to protect their rights. At initial evaluation, 39/48 (81.3%) participants were well-acquainted with an informed consent form (ICF) and answered “ICF gives important information, including possible risks and benefits, about a medical procedure or treatment, genetic testing, or a clinical trial.” The response improved to 47/48 (98.0%) post-evaluation showing a statistically significant response improvement of 16.7% (p = 0.008) (Table 1). The disagreement rate with the statement “once enrolled in a clinical research study, participants must complete the study” increased to about 41/48 (85.4%) from the initial 30/48 (62.5%), reflecting a statistically significant response improvement of 22.9% (p = 0.001) (Table 1). During pre-evaluation, participants when asked “In case of an injury or death after participation in Clinical Trial, the participant should receive…,” 31/48 (64.6%) correctly answered “medical support” and “justified compensation.” Subsequent post-evaluation showed a marginal increase to 36/48 (75.0%), with a response improvement of 10.4% (p = 0.063). Post-evaluation, approximately 40/48 (83.4%) participants affirmed their disagreement with the statement “Clinical Trial can never be safe for patients as a new drug is tested,” showing a marginal increase from the initial 35/48 (72.9%) (improvement of 10.5%) (p = 0.063) (Table 1).

**Table 1:** Pre and Post-evaluation of participants attending the PACER workshop 1: Awareness of clinical research. *: denotes participants who had undertaken the online PACER course pre-evaluation. PACER = Patient Advocates for Clinical Research

Questions	Responses	Pre-workshop*		Post-workshop	
		Number of Participants (n = 48)	Percent (%)	Number of Participants (n = 48)	Percent (%)
What is the full form of PACER?	Patient Advocates for Clinical Research Engineers and Report Frameworks	1	2.1%	1	2.1%
	Patient Advocates for Clinical Research	44	91.7%	46	95.9%
	Public Advocates for Clinical Reports Empowerment and Regulatory Frameworks	0	0.0%	1	2.1%
	Patient Advocates for Clinical Research Engagement and Reports Frameworks	3	6.3%	0	0.0%
	I don’t know	0	0.0%	0	0.0%
What is clinical research?	Clinical research is just an experiment where the patient needs to give blood for testing	1	2.1%	1	2.1%
	Clinical research is a long-term study that involves psychological tests	0	0.0%	1	2.1%
	Clinical research helps find new and better ways to detect, diagnose, treat, and prevent diseases	47	97.9%	46	95.9%
	Clinical research is a study of family history that involves talking to family members to learn about people’s medical needs and history	0	0.0%	0	0.0%
	I don’t know	0	0.0%	0	0.0%
Patient Advocacy Groups (PAGs)	PAGs are lawyers who help patients file their cases	0	0.0%	0	0.0%
	PAGs educate patients about their legal rights in clinical research	6	12.5%	3	6.3%
	PAGs help patients communicate with their healthcare providers so they get the information they need to make decisions	39	81.3%	42	87.5%
	PAGs help patients get compensation after participation in research	2	4.2%	3	6.3%
	I don’t know	1	2.1%	0	0.0%
Before enrolment of participants in a clinical trial, which document is given to participants for understanding the study?	Protocol	3	6.3%	0	0.0%
	Patient Information Sheet	4	8.3%	5	10.4%
	Informed Consent Form	6	12.5%	7	14.6%
	Both b and c	35	72.9%	36	75.0%
	I don’t know	0	0.0%	0	0.0%
Informed Consent Form (ICF)	ICF is a lengthy form for patients enrolling in clinical research	0	0.0%	0	0.0%
	ICF gives important information, including possible risks and benefits, about a medical procedure or treatment, genetic testing, or a clinical trial	39	81.3%	47	98.0%
	ICF provides information only about the medicine used in clinical research	2	4.2%	0	0.0%
	ICF informs patients that they are participating in clinical research which will benefit them	6	12.5%	1	2.1%
	I don’t know	1	2.1%	0	0.0%
Once enrolled in clinical research, study participants need to complete the study	True	14	29.2%	5	10.4%
	False	30	62.5%	41	85.4%
	I don’t know	4	8.3%	2	4.2%
In case of injury or death after participation in a clinical trial, participants should receive ______.	Emotional support	1	2.1%	1	2.1%
	Medical support	1	2.1%	1	2.1%
	Justified compensation	11	22.9%	10	20.8%
	Both b and c	31	64.6%	36	75.0%
	I don’t know	4	8.3%	0	0.0%
Clinical trials can never be safe for patients as a new drug is tested	True	8	16.7%	6	12.5%
	False	35	72.9%	40	83.4%
	I don’t know	5	10.5%	2	4.2%
Principal Investigator (Research Doctor)	The Principal Investigator (Research Doctor) never gives full information about the clinical research	2	4.2%	1	2.1%
	The Principal Investigator (Research Doctor) administers the informed consent process and answers each query related to the patient’s participation	40	83.3%	46	95.9%
	The Principal Investigator (Research Doctor) only talks about the benefits of the research	4	8.4%	1	2.1%
	The Principal Investigator (Research Doctor) asks to sign the ICF	1	2.1%	0	0.0%
	I don’t know	1	2.1%	0	0.0%
Principal Investigator (Research Doctor) needs to get approval from which of the following before initiating the clinical trial/study at the hospital site?	Quality Department	1	2.1%	0	0.0%
	Ethics Committee	30	62.5%	35	72.9%
	Health Authority of India	7	14.6%	11	22.9%
	Hospital Head	4	8.3%	2	4.2%
	I don’t know	6	12.5%	0	0.0%

Patient awareness about the research conduct and the team involved is essential for their comprehension of the study. In the post-evaluation, 46/48 (95.9%) participants affirmed their agreement with the statement suggesting that the “Principal Investigator (Research Doctor) managed the Informed Consent Process and addressed all patient queries,” which significantly increased from the initial 40/48 (83.3%) (12.6% improvement) (p = 0.031) (Table 1). Post-assessment, 35/48 (72.9%) participants unmistakably identified the “Ethics Committee” correctly when asked “Principal Investigator (Research Doctor) needs to take approval from which of the following before initiating the clinical trial/study at the hospital site,” compared to the pre-evaluation response by 30/48 (62.5%) (Table 1). A marginal response improvement of 10.4% (p = 0.063) was seen between pre- and post-evaluation. An overall improvement of 9.4% (𝜒^2^ = 46.173; p < 0.001) was seen in response to the conduct of the workshop among participants about clinical research and its conduct.

The second workshop emphasized the GCP and research ethics and the processes in place to uphold ethical principles in clinical research activities. The workshop was held on June 15, 2023, and was attended by 45 participants.

GCP represents the minimum standards in science, ethics, and quality, and plays a pivotal role in safeguarding participants and maintaining data integrity. Therefore, there is a basic need to educate patients keen on engaging in clinical research and trials. Initially, 37/45 (82.2%) participants were familiar with the term “Good Clinical Practices” which raised to 42/45 (93.3%) after evaluation, i.e., an improvement of 11.1% (p = 0.125) (Table 2). After evaluation, 42/45 (93.3%) participants held the opinion that “GCP offers public assurance by safeguarding the rights, safety, and wellbeing of research participants and ensuring the reliability of research data,” signifying a noteworthy rise from the initial 34/45 (75.6%) in pre-evaluation and a statistically significant response improvement of 17.7% (p = 0.008) (Table 2).

**Table 2:** Pre and post-evaluation of participants attending the PACER workshop 2: GCP and research ethics. **: denotes participants had undertaken the online PACER course and workshop 1 pre-evaluation. PACER = Patient Advocates for Clinical Research; GCP = Good Clinical Practice

Questions	Responses	Pre-workshop**		Post-workshop	
		Number of Participants (n = 45)	Percent (%)	Number of Participants (n = 45)	Percent (%)
What is the full form of PACER?	Patient Advocates for Clinical Research Engineers and Report Frameworks	2	4.4%	0	0.0%
	Patient Advocates for Clinical Research	42	93.3%	43	95.6%
	Public Advocates for Clinical Reports Empowerment and Regulatory Frameworks	0	0.0%	0	0.0%
	Patient Advocates for Clinical Research Engagement and Reports Frameworks	1	2.2%	1	2.2%
	I don’t know	0	0.0%	1	2.2%
What is the full form of GCP?	Good Clinical Practice	37	82.2%	42	93.3%
	Google Cloud Program	3	6.7%	0	0.0%
	Good Clinical Program	1	2.2%	1	2.2%
	Global Clinical Practice	4	8.9%	1	2.2%
	I don’t know	0	0.0%	1	2.2%
GCP is essential for designing, conducting, performing, monitoring, auditing, recording, analyzing, and reporting clinical trials	True	43	95.6%	44	97.8%
	False	1	2.2%	1	2.2%
	I don’t know	1	2.2%	0	0%
GCP provides public assurance that	The rights and safety of the public are protected	3	6.7%	0	0.0%
	The rights, safety, and well-being of research participants are protected and that research data are reliable	34	75.6%	42	93.3%
	Results are reliable	0	0.0%	0	0.0%
	Safety of participants is observed and research results are reliable and protected	7	15.6%	2	4.4%
	I don’t know	1	2.2%	1	2.2%
What is the role of an Ethics Committee?	To design the protocol for a clinical trial	6	13.3%	5	2.2%
	To assess whether a clinical trial is ethical to perform in the given subject population	32	71.1%	36	80.0%
	To analyze the data from a clinical trial	3	6.7%	2	4.4%
	To assess whether a medicinal product should be granted a marketing authorization	2	4.4%	2	11.1%
	I don’t know	2	4.4%	0	0.%
Ethical considerations include	Respect	1	2.2%	0	0.0%
	Justice	0	0.0%	1	2.2%
	Beneficence and non-maleficence	1	2.2%	0	0.0%
	All of the above	43	95.6%	44	97.8%
	I don’t know	0	0.0%	0	0.0%
Ethics Committee quorum requires at least _____members in a meeting room for reviewing a research study	At least 7 members	16	35.6%	11	24.4%
	At least 10 members	2	4.4%	0	0.0%
	At least 5 members	21	46.7%	32	71.1%
	At least 15 members	0	0.0%	0	0.0%
	I don’t know	6	13.3%	2	4.4%
Research protocol, patient information sheet, and the informed consent form should be approved by the Ethics Committee	True	42	93.3%	44	97.8%
	False	3	6.7%	1	2.2%
	I don’t know	0	0.0%	0	0.0%
Patient-centered care can improve patient outcomes and can provide patient satisfaction	True	38	84.4%	40	88.9%
	False	5	11.1%	2	4.4%
	I don’t know	2	4.4%	3	6.7%
Patient Advocacy Groups (PAGs) can	Uphold ethical principles	2	4.4%	4	8.9%
	Promote trust among stakeholders	3	6.7%	1	2.2%
	Both a and b	38	84.4%	40	88.9%
	None	0	0.0%	0	0.0%
	I don’t know	2	4.4%	0	0.0%

The ethics committee, as an independent entity, ensures that the study team adheres to GCP guidelines and safeguards the safety and welfare of participants in a clinical trial. Patient awareness regarding the ethics committee’s function is crucial to ensure the ethical consideration and implementation of their rights. At the initial evaluation, 32/45 (71.1%) participants identified the role of an Ethics Committee as “assessing whether a clinical trial is ethical to conduct in the given subject population” which increased to 36/45 (80%) at post-evaluation showing a response improvement to 8.9% (p = 0.344) (Table 2). Post-evaluation, the awareness that the “Ethics Committee requires a minimum of five members present in a meeting room for reviewing a research study” notably increased to 32/45 (71.1%) from the initial 21/45 (46.7%) showing a statistically significant response improvement of 24.4% (p = 0.001) (Table 2).

The evolution of the healthcare system to patient-centered care highlights the necessity to educate patients about their engagement in patient-centered research. Post-assessment of participants on patient-centered care and contributions of PAGs revealed a response improvement of 4.5% (p = 0.625) in comparison to pre-assessment (40/45 (88.9%) versus 38/45 (84.4%)) (Table 2). An overall improvement of 8.2% (𝜒^2^ = 25.412; p < 0.001) was significantly seen among participants in response to the conduct of the workshop. Moreover, in the workshop’s interactive session, patients shared their experiences about being involved in trials and were interested to know more about the “recruitment procedure,” “randomization,” “placebo,” and “ethical rights as a participant.”

The FGD was an online event, attended by 140 participants including parents, patients, survivors, and patient advocates who raised concerns about a lack of understanding of “what clinical research is.” Grievances were raised about unethical recruitment approaches by the study investigators, which they felt could be countered by transparency and communication. Participants highlighted the need for the readability of patient information sheets and clarity on consent forms. They also felt that the disease biology and research methods used must be shared in simpler language. The participants wanted to know “What is an ethics committee?,” “Do they have any training?,” and “Can they join the ethics committee?.” Participants also wanted to know “What is clinical trial insurance and compensation and if it will impact their personal health insurance?.” Participants opened up about their fear surrounding the idea of participation in clinical trials due to their elementary understanding of clinical research especially in a hospital environment.

## Discussion

In this study, while the online self-paced educational program was a successful approach for the preliminary engagement of patients, survivors, and PAGs, the need for an in-person forum to discuss and review case studies in a group was equally critical.

The faculty of the online program and the workshops were carefully chosen to be comfortable with simple language communication and were both patient advocates and clinical researchers or ethicists.

We feel that the overall improvement in awareness among the participants about clinical research, GCP, research ethics, and the role of patient advocates in clinical research was due to the engagement that the speakers/trainers were able to create with the PAG. The participants were also comfortable voicing their concerns in their peer group.

In recent years, there has been a push to include patients and advocates in the identification of clinical research priorities, leading and designing clinical trials and real-world evidence studies, participating in research ethics committees, and improving patient access to clinical trials. Despite efforts to involve patients and advocates in clinical research influencing their care, there is considerable ground to cover for truly inclusive participation in research. India and other developing countries have even further to go in actively incorporating patients and advocates throughout the research continuum [35].

Patients and advocates, although well-versed in their personal condition, do not always have a broader knowledge of the disease or an understanding of the research process. They require supplementary information and guidance to actively participate in research with a thorough understanding. These initial barriers must be overcome to establish meaningful and collaborative relationships. Researchers frequently engage with clinicians involved in treating the diseases they study and join grand rounds and clinical seminar series to grasp how these diseases are managed clinically. Researchers must contribute to educating patients and advocates, enabling them to be well-informed and capable of educating others within the research community.

In India, events such as International Clinical Trials (ICT) day offer an opportunity for clinicians, researchers, patients, and patient advocates to engage and get a glimpse into the exciting research occurring locally. Stakeholders such as national regulatory bodies, ethics committees, academics, contract research organizations, sponsors, and patients collaborated on the ICT day celebration at the Indian Society of Council Research, highlighting the stakeholders’ challenges, unaddressed areas in new regulations, and patients insights on clinical trial participation benefits [36]. Many national societies and PAGs have recognized the educational needs of patients and patient advocates and structured programs according to the requirements [37].

CTTI recommends best practices for effective engagement with patient groups around clinical trials that focus on proactive identification, engagement, and bringing the patient voices to stakeholders. Patients and advocates can also help by establishing policies that require full disclosure, transparency, and accountability [38,39].

The shift toward patient-centered medicine is beginning to enable patients to have a voice in their healthcare decisions. However, a similar pace of change is not mirrored in clinical research where patients are often the subjects rather than actively engaged participants. Targeted initiatives designed for enhancing patient involvement may include (1) identifying research priorities from the patient’s perspective, (2) involving patient associations in leading and designing research, (3) improving access to clinical trials, (4) availability of appropriate and complete information about the study, (5) evaluation of patients’ experience as participants, and (6) dissemination of study findings to the participants and its applicability [18]. Achieving these objectives requires transitioning from the conventional beneficence-driven model to autonomy-focused models which will be important in fostering the growth of informed and “expert” patients [18].

The public’s limited understanding of research, especially its sometimes ambiguous and technical nature, fosters suspicion and fear. Lack of information about critical parameters such as compensation, confidentiality, and data availability creates further distrust. Patients, their families, caregivers, advocacy groups, and the public should be kept informed not only of the specific protocols they contribute to but also of the entire clinical research process for ethical, methodological, and operational reasons. Collaborative efforts among healthcare professionals, media, patient groups, and pharmaceutical companies are important for providing accurate information and research goals clarification and safeguarding participant rights. Society must recognize that as research drives progress, it is critical to emphasize “expert” patient involvement and acknowledge previous trial participants for past breakthroughs.

Public awareness programs are a vital first step to collaborative patient-centered research. These efforts must be engaging and responsive to the ground-level situation and should inform local research policies and processes and address misconceptions if any. Larger, cross-cultural surveys are needed for broader insights into such endeavors [27-31].

The web pages of regulatory agencies such as the US Food and Drug Administration [40], national health institutes, such as the NIH in the United States [41], and scientific associations such as the American Society of Clinical Oncology [42] include patient-directed information on the basic principles of clinical research. Similarly, some very interesting initiatives have emerged, such as Health Talk Online [43], supported by Oxford University, that include videos in which actual patients relate their experiences as clinical trial participants and elucidate fundamental elements within the research process. Second, efforts have been oriented toward empowering patients to be experts through tailored training courses and educational programs [44]. The educational program offered by the European Patients’ Academy on Therapeutic Innovation is an alliance of 30 European organizations dedicated to informing individuals affected by various diseases about medical research. This expert course comprehensively covers the clinical trial process, encompassing the following six training modules: (1) discovery of medicines and planning of medicine development; (2) non-clinical testing and pharmaceutical development; (3) exploratory and confirmatory clinical development; (4) clinical trials; (5) regulatory affairs, medicinal product safety, pharmacovigilance, and pharmacoepidemiology; and (6) health technology assessment principles and practice. Its objective is to empower patients and their families to actively engage in the research process by collaborating directly with industry, regulatory bodies, or patient associations [45-47].

In India, an overwhelming majority of the public endorsed research goals and benefits (94.1% stated that research benefits society) during the PARTAKE survey [32], yet there was a trust deficit. The situation may have changed slightly with awareness and trust marginally increasing after COVID-19 vaccine trials in India. A cross-sectional survey across Delhi National Capital Region post-COVID-19 pandemic reflected an increase in awareness about clinical research/clinical trials to 87% compared to earlier surveys in Delhi (26%), Pune (25%), and Mumbai (52.2%) [48]. Yet, there is a long way to go for PACER, a pioneering initiative in India, aimed at raising patient awareness and educating patients as experts to empower them toward informed participation in clinical research. Targeting PAGs rather than individual patients for awareness of clinical research is essential as they have a broader reach and can disseminate information to a larger audience of patients and caregivers with greater trust and cohesion. PAGs can work to ensure that research studies are patient-centered, ethical, and inclusive of patients’ interests.

Limitations

The study is focused on PAGs having a high interest in clinical research and may not represent the general population, leading to self-selection bias. The evaluation of knowledge improvement was conducted immediately after the workshops and courses. Therefore, the retention of knowledge and its implication in practice by the participants needs follow-up studies. A follow-up evaluation with the participants will aid in understanding the success of the workshops. The workshops mainly covered important aspects such as informed consent, ethics committees, and patient-centered research. Other relevant topics such as data privacy and adverse event reporting were not covered which could have provided a comprehensive understanding of clinical research.

## Conclusions

The PACER initiative through its multimodal and tiered methodology is a step toward information and education of PAGs regarding clinical research. It will empower patients, caregivers, and survivors and create awareness of patients’ rights in research. There is a need for such initiatives to increase awareness of clinical research and a significant step toward creating an “expert” patient.
